# Supplementary figures and images for: Insights into the genome of Methylobacterium sp. NMS14P, a novel bacterium for growth promotion of maize, chili, and sugarcane
Source: PLoS One. 2023 Feb 7;18(2):e0281505. doi: 10.1371/journal.pone.0281505 (PMC9904496; doi:10.1371/journal.pone.0281505)

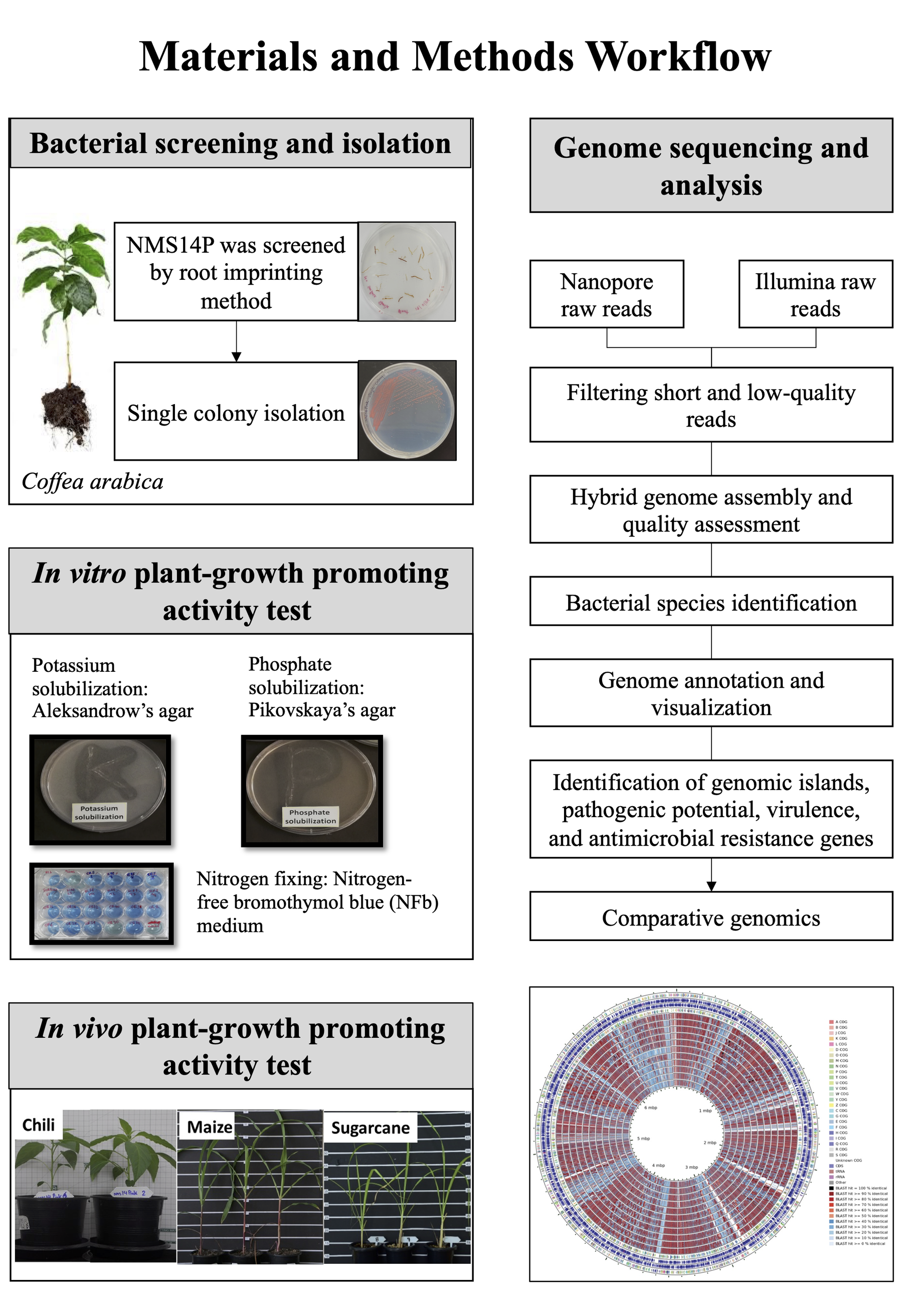

Supplement: S1 Fig — (TIF) [file pone.0281505.s001.tif]

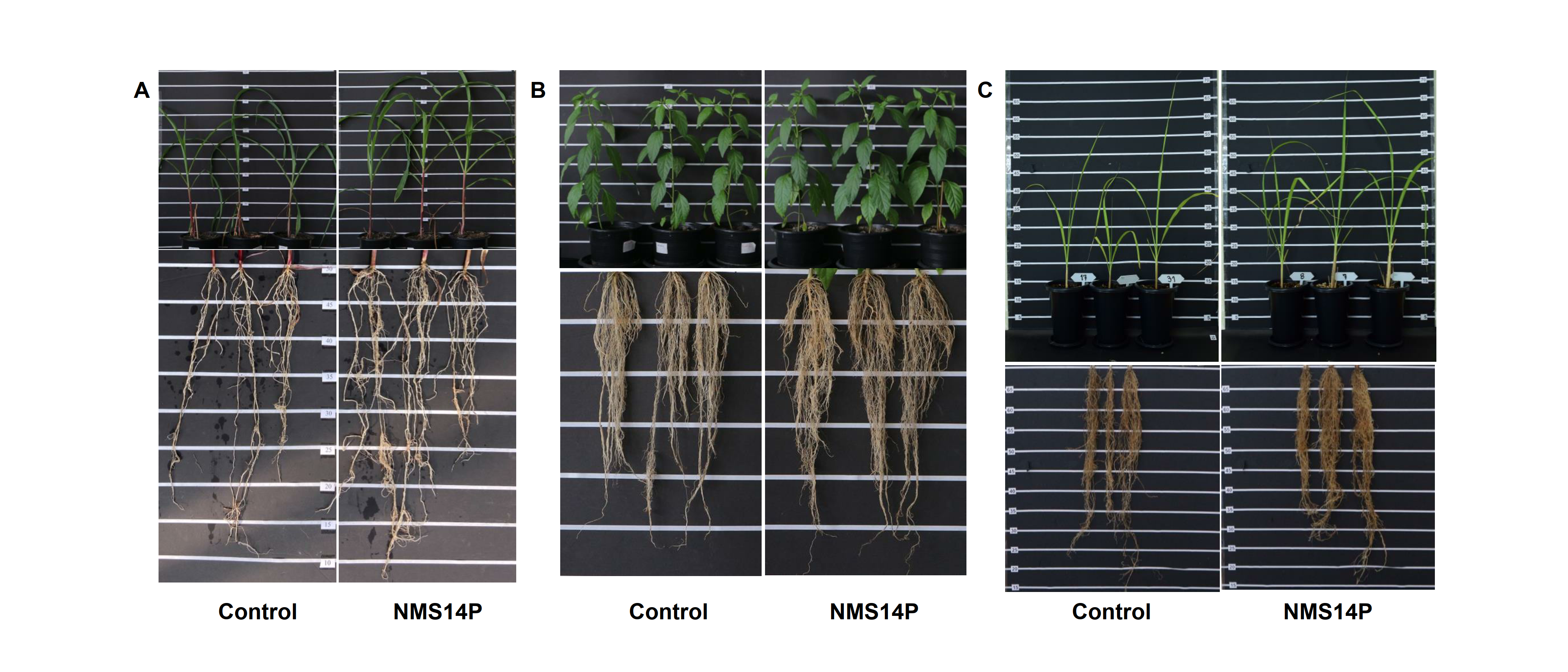

Supplement: S2 Fig — Comparison of plant biomass of treated- and control (A) maize, (B) chili, and (C) sugarcane at 35-, 75-, and 56-day post-inoculation, respectively. (TIF) [file pone.0281505.s002.tif]

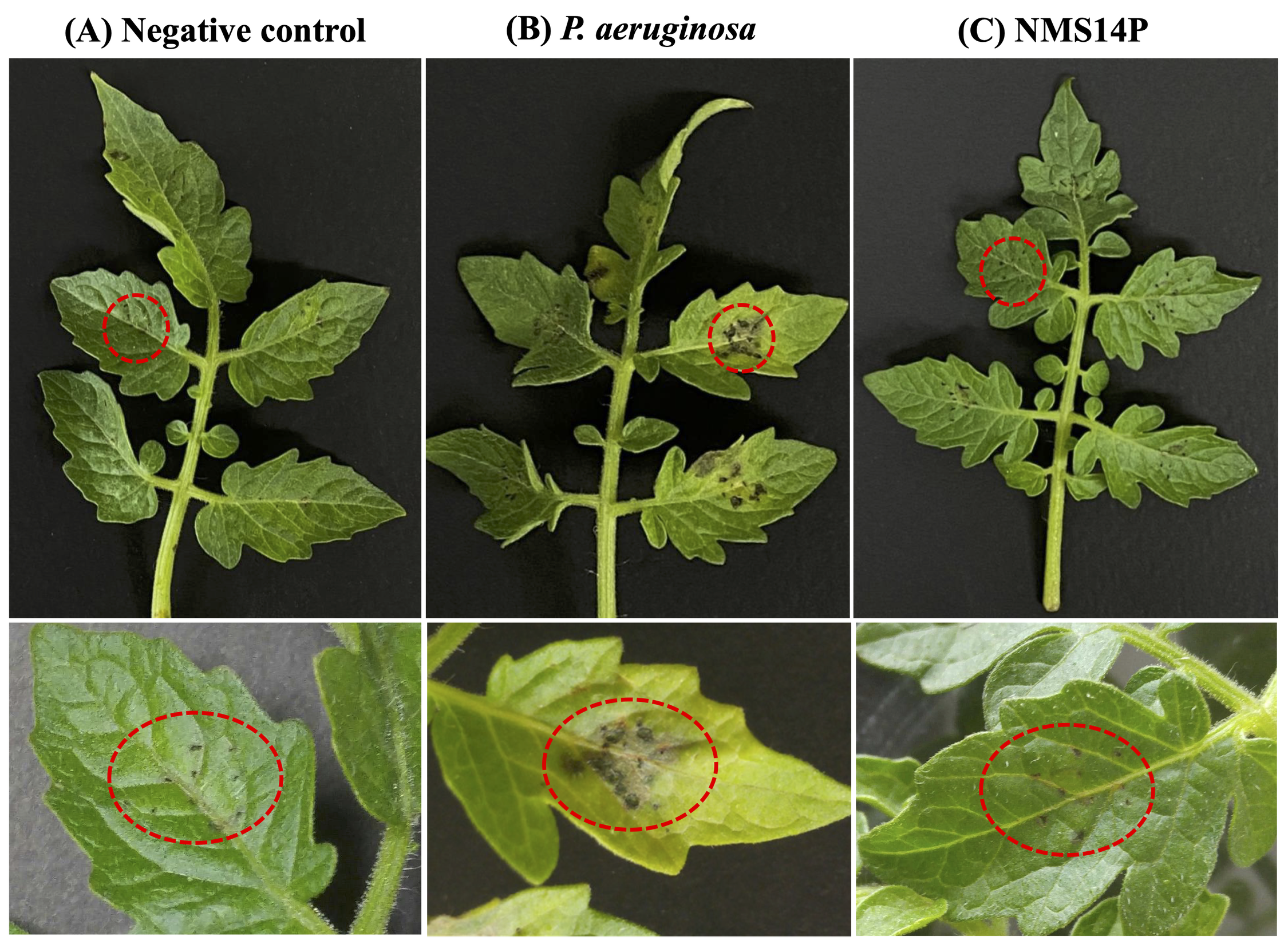

Supplement: S3 Fig — The hypersensitive response of tomatoes (Solanum lycopersicum L.) infiltrated with (A) sterile distilled water, (B) Pseudomonas aeruginosa, and (C) NMS14P. (TIF) [file pone.0281505.s003.tif]

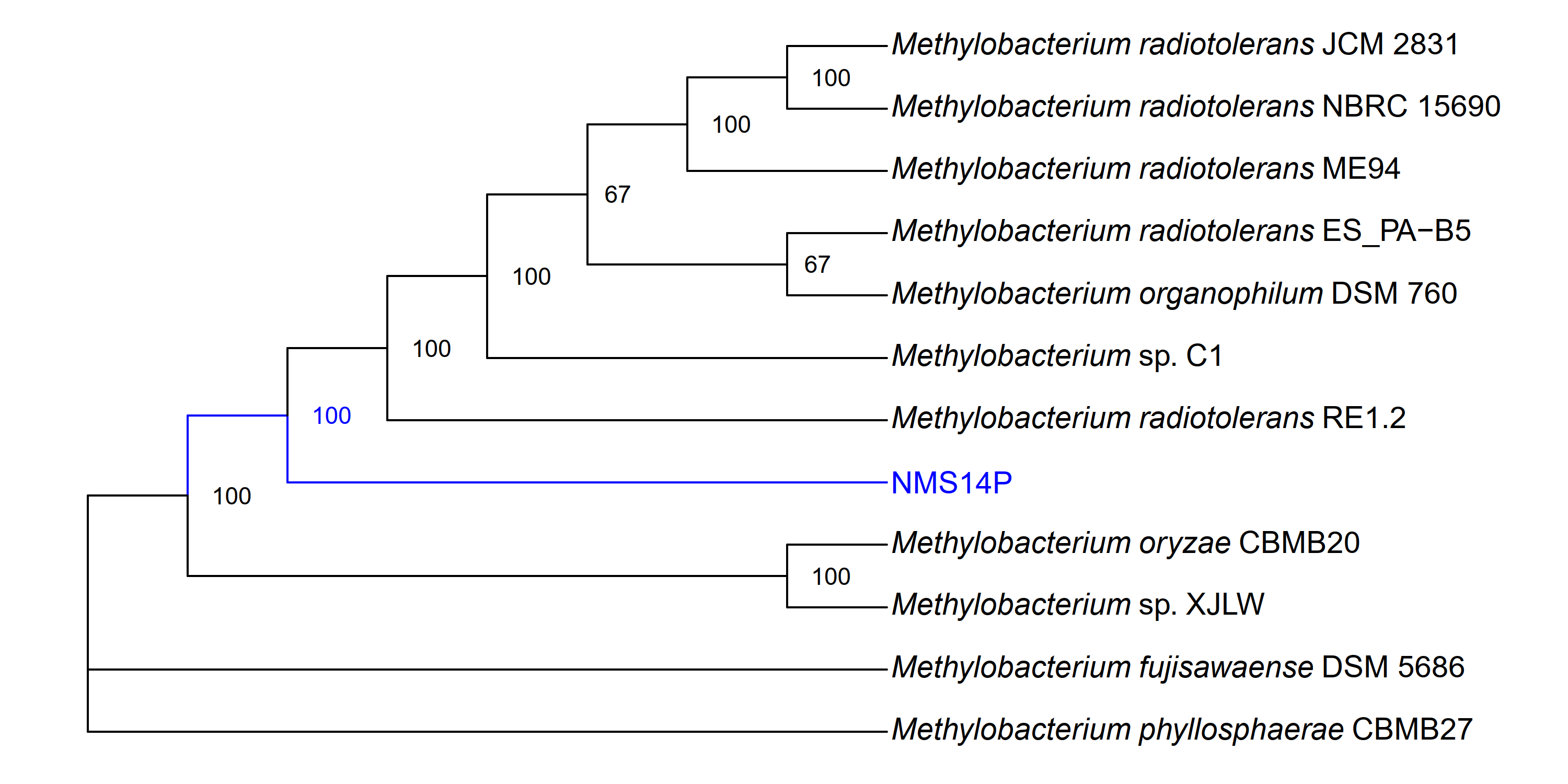

Supplement: S4 Fig — (TIF) [file pone.0281505.s004.tif]

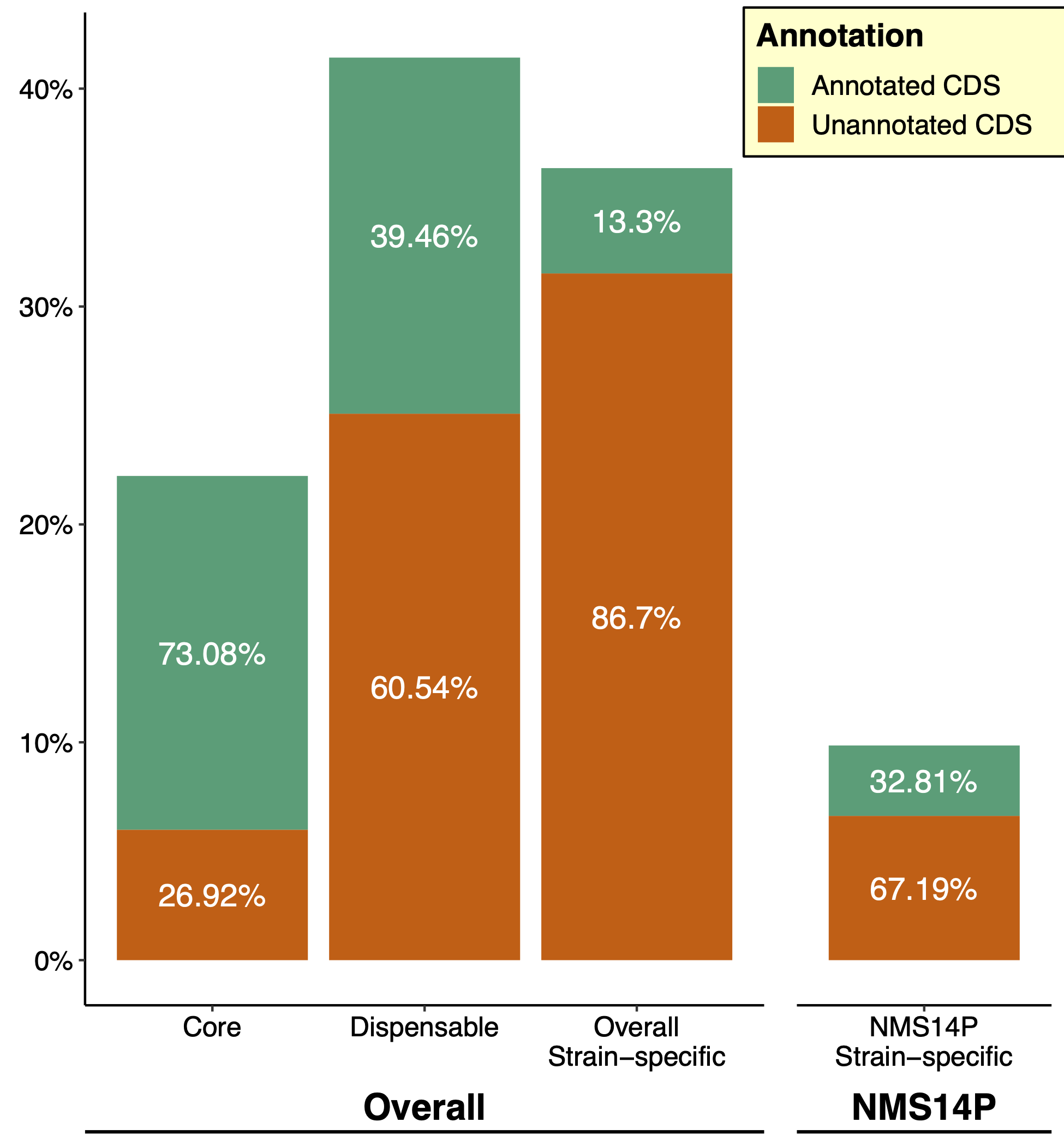

Supplement: S5 Fig — (TIF) [file pone.0281505.s005.tif]

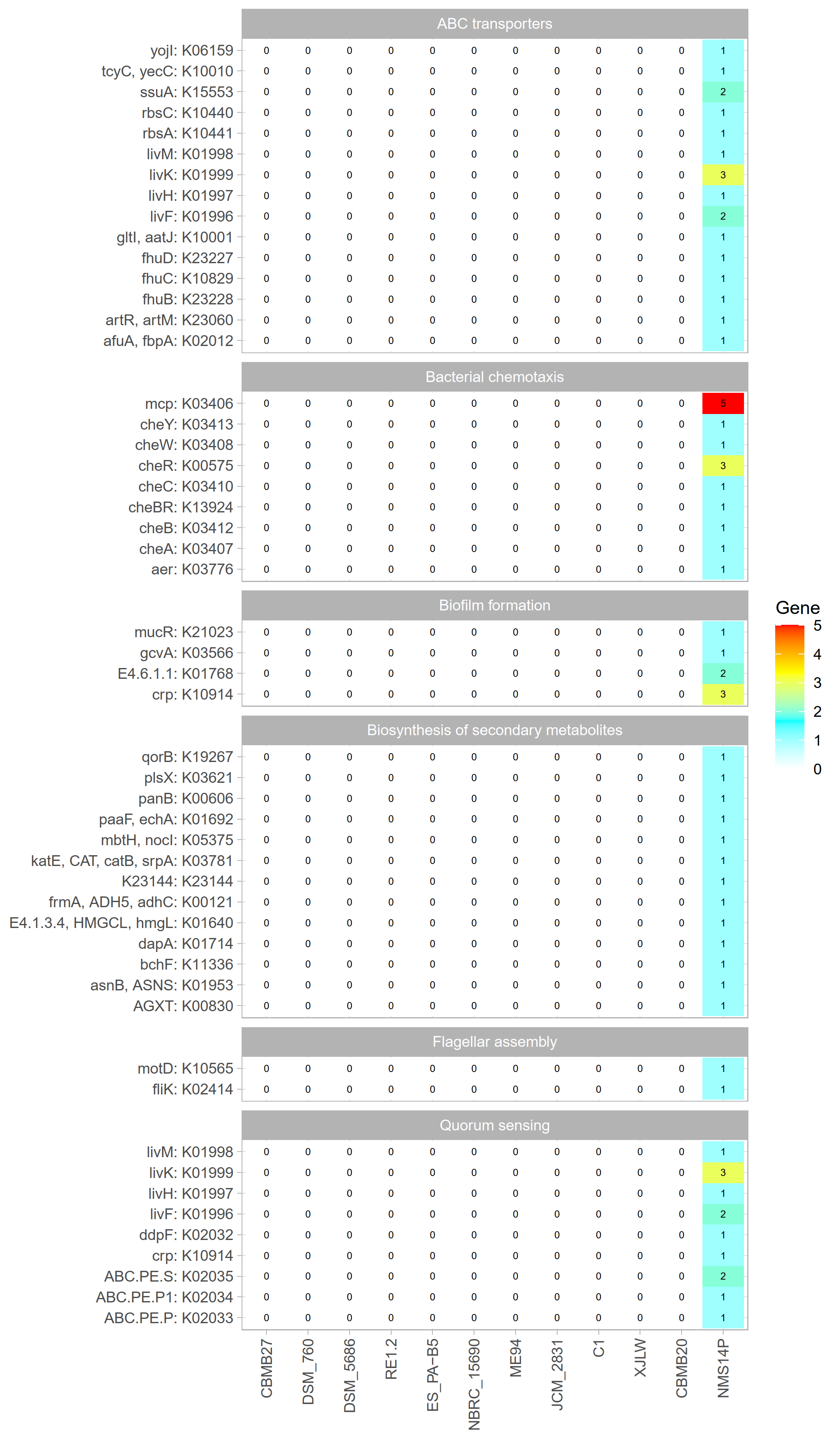

Supplement: S6 Fig — (TIF) [file pone.0281505.s006.tif]

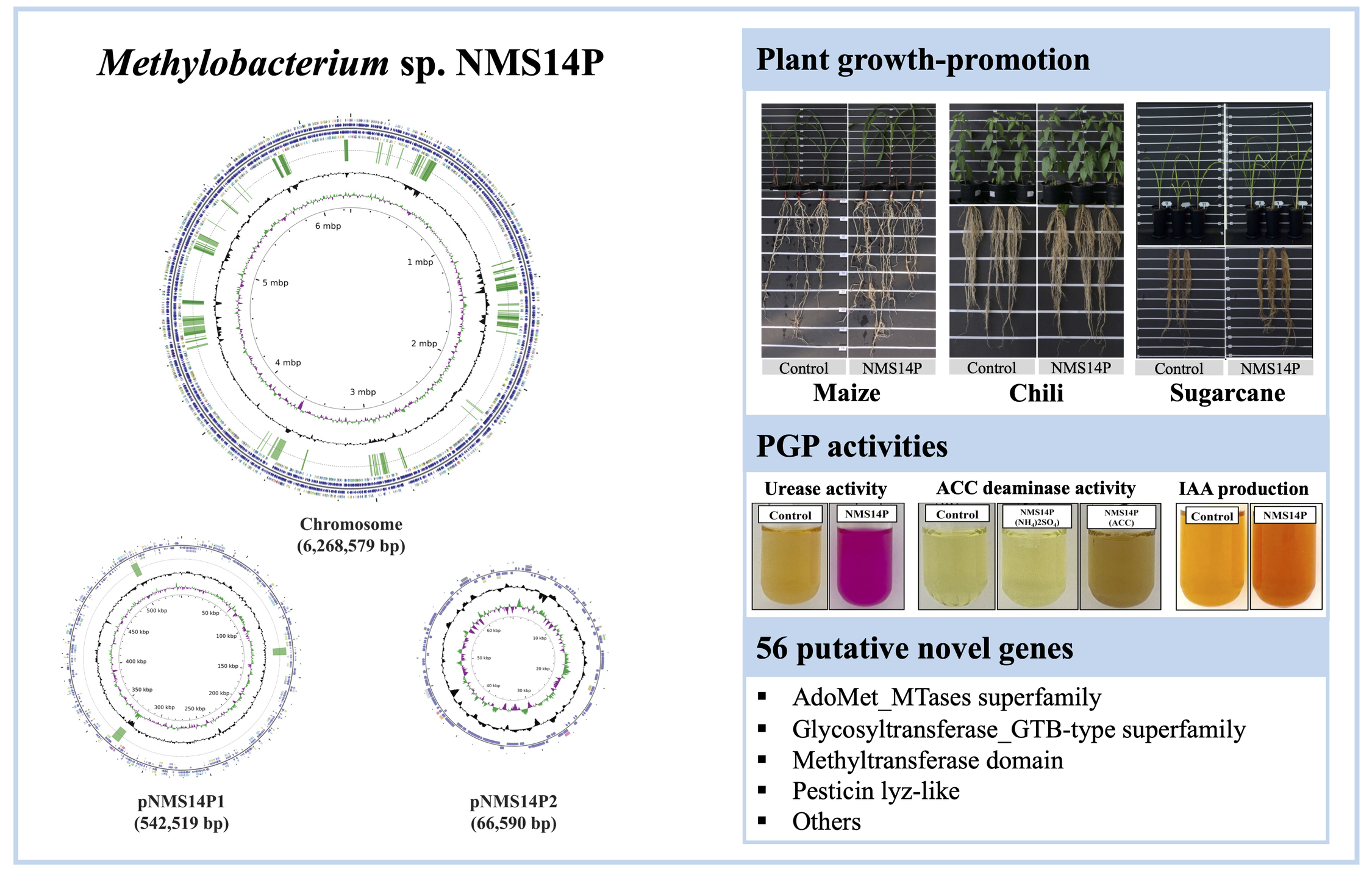

Supplement: S1 Graphical abstract — (TIF) [file pone.0281505.s017.tif]
