# Supplementary material for: Insights into the genome of Methylobacterium sp. NMS14P, a novel bacterium for growth promotion of maize, chili, and sugarcane
Source: PLoS One. 2023 Feb 7;18(2):e0281505. doi: 10.1371/journal.pone.0281505 (PMC9904496; doi:10.1371/journal.pone.0281505)
Supplement: S1 Text — (DOCX) [file pone.0281505.s016.docx]

**S1 Text. Pairwise alignment of Sanger-derived 16S rRNA sequence with 16S rRNA sequences extracted from the genome assembly.**

>Sanger-derived 16S rRNA read sequenced using the 1492R primer

NNNNNNGNNCTACCGTGGTCGCCTGCCTCCTTGCGGTTGGCGCAGCGCCGTCGGGTAAGACCAACTCCCATGGTGTGACGGGCGGTGTGTACAAGGCCCGGGAACGTATTCACCGTGGCATGCTGATCCACGATTACTAGCGATTCCGCCTTCATGCACTCGAGTTGCAGAGTGCAATCCGAACTGAGACGGCTTTTGGGGATTTGCTCCAGATCGCTCCTTCGCGTCCCACTGTCACCGCCATTGTAGCACGTGTGTAGCCCATCCCGTAAGGGCCATGAGGACTTGACGTCATCCACACCTTCCTCGCGGCTTATCACCGGCAGTCTCCCTAGAGTGCCCAACTGAATGATGGCAACTAAGGACGTGGGTTGCGCTCGTTGCGGGACTTAACCCAACATCTCACGACACGAGCTGACGACAGCCATGCAGCACCTGTGTGCGCGCCACCGAAGTGGACCCCAAATCTCTCTGGGTAACACGCCATGTCAAAGGATGGTAAGGTTCTGCGCGTTGCTTCGAATTAAACCACATGCTCCACCGCTTGTGCGGGCCCCCGTCAATTCCTTTGAGTTTTAATCTTGCGACCGTACTCCCCAGGCGGAATGCTCAAAGCGTTAGCTGCGCTACTGCGGTGCAAGCACCCCAACAGCTGGCATTCATCGTTTACGGCGTGGACTACCAGGGTATCTAATCCTGTTTGCTCCCCACGCTTTCGCGCCTCAGCGTCAGTAATGGTCCAGTTGGCCGCCTTCGCCACCGGTGTTCTTGCGAATATCTACGAATTTCACCTCTACACTCGCAGTTCCACCAACCTCTACCATACTCAAGCGTCCCAGTATCGAAGGCCATTCTGTGGTTGAGCCACAGGCTTTCACCCCCGACTTAAAACGCCGCCTACGCGCCCTTTACGCCCAGTGATTCCGAGCAACGCTAGCCCCCTTCGTATTACCGCGGCTGCTGGCACGAAGTTAGCCGGGGCTTATTCCTCCGGTACCGTCATTATCGTCCCGGATAAAAGAGCTTTACAACCCTAAGGCCTTCATCACTCACGCGGCATGGCTGGATCAGGCTTGCGCCCATTGTCCAATATTCCCCACTGCTGCCTCCCGTAGGAATCTGGGCCGGGTCTCAGTCCCAGTGTGGCTGATCATCCTCTCAAACCACCTACTGATCGTCGCCTTGGTAGGCATTACCNNNCCAACTAGTTAATCAAACGGGGGCGATTTTCCGGCAATAAACTTTTCCCCAAAAGGGCTNNCCGGTNNNNCCCTATTTTCCCGGGTTTTTCCAANCCAAAGGGCNTTTCCCCNNTTATTNCCCNNNCCCCTTAACCCAAAGGGCCCCCCCATTNNNGGGTTAAACCNNCCCCGNNNTCCCCACNNAGNNNGCNAATTNNAANNAAAA

**Note:** Bases with red highlights were trimmed due to the low quality and/or the presence of N bases. The original file (.ab1 format) of the Sanger sequencing result can be downloaded through the following link: <https://github.com/NuruddinKhoiry/NMS14P>

Pairwise alignment of Sanger sequencing-derived 16S rRNA sequence with five copies of 16S rRNA sequences of NMS14P extracted from the genome assembly was performed using BLASTn and MUSCLE. The pairwise alignment results are shown in Table 1 and Fig 1.

**Table 1.** Pairwise alignment of Sanger-derived 16S rRNA sequence with 16S rRNA sequences extracted from the genome assembly with BLASTn.

| Subject | Query | Max score | Total score | Query coverage | E value | Percent identity |
| --- | --- | --- | --- | --- | --- | --- |
| NMSCH_06308 16S rRNA | Sanger 16S rRNA | 2167 | 2167 | 100% | 00.00 | 99.58 |
| NMSCH_03727 16S rRNA | Sanger 16S rRNA | 2167 | 2167 | 100% | 00.00 | 99.58 |
| NMSCH_02591 16S rRNA | Sanger 16S rRNA | 2167 | 2167 | 100% | 00.00 | 99.58 |
| NMSCH_01635 16S rRNA | Sanger 16S rRNA | 2167 | 2167 | 100% | 00.00 | 99.58 |
| NMSCH_04776 16S rRNA | Sanger 16S rRNA | 2161 | 2161 | 100% | 00.00 | 99.50 |

**
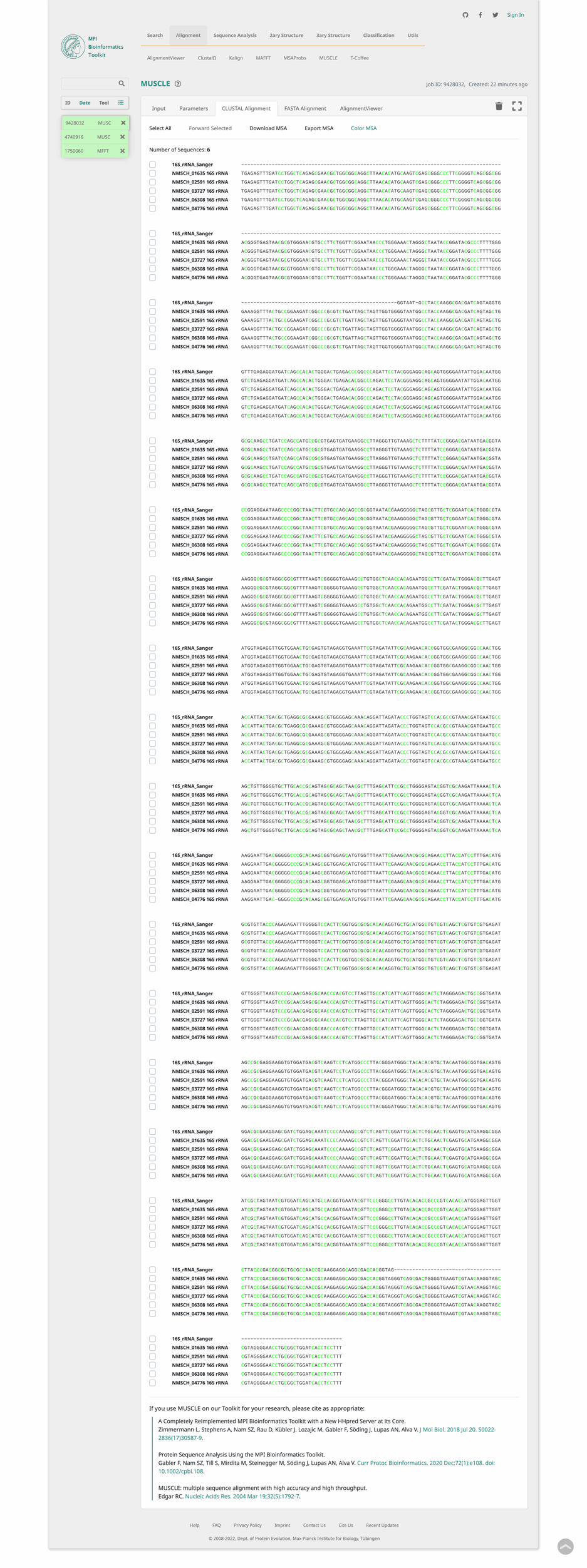
**

**Fig 1. Pairwise alignment of Sanger-derived 16S rRNA sequence with 16S rRNA sequences extracted from the genome assembly**
