# Supplementary material for: Insights into the genome of Methylobacterium sp. NMS14P, a novel bacterium for growth promotion of maize, chili, and sugarcane
Source: PLoS One. 2023 Feb 7;18(2):e0281505. doi: 10.1371/journal.pone.0281505 (PMC9904496; doi:10.1371/journal.pone.0281505)
Supplement: Attachment — Submitted filename: R Report_02.07.2022.pdf [file pone.0281505.s018.pdf]

Reviewer: 1

Recommendation: Publish after major revisions.

Comments:
This manuscript presents a study on Insights into the genome of a novel *Methylobacterium* sp. NMS14P, a broad host range plant growth-promoting bacterium. Moreover, the study suffers from several shortfalls including poor presentation. As the comments are numerous to list here, I had marked most of my comments directly on the annotated manuscript.
Comment 1: The first and foremost concern of the study is that the title does not adequately reflect the content of the manuscript. For example, the study about the effects of *Methylobacterium* sp. NMS14P inoculation on the growth of maize (*Zea mays*), chili (*Capsicum annuum*), and sugarcane (*Saccharum officinarum* L.) is not reflected in the title.

Comment 2: Should be add one paragraph about maize (*Zea mays*), chili (*Capsicum annuum*), and sugarcane (*Saccharum officinarum* L.) in introduction section.

Comment 3: The study has no mechanistic hypothesis.

Comment 4, Line 104: Nutrient broth medium compositions should be written

Comment 5: Much important information is missing in the methods section. The type of soil, soil physicochemical characteristics, variety of maize, chili and sugarcane, plant growth conditions (light, temperature, etc.) are missing.

Comment 6: Discussion should be improved. Why *Methylobacterium* sp. NMS14P has these positive effects, in which particular part or mechanism is affecting?
