# Supplementary material for: Insights into the genome of Methylobacterium sp. NMS14P, a novel bacterium for growth promotion of maize, chili, and sugarcane
Source: PLoS One. 2023 Feb 7;18(2):e0281505. doi: 10.1371/journal.pone.0281505 (PMC9904496; doi:10.1371/journal.pone.0281505)
Supplement: Attachment — Submitted filename: Response to Reviewers.docx [file pone.0281505.s020.docx]

- **Response to the Reviewer #1**

**Reviewer #1: Dear authors**

Thanks for your answers. However, one of the most important features of this work still requires further validation. As I previously addressed in my comments, the authors should provide HR assay output for the studied strains. Because these strains have the potential to introduce into agricultural markets by the means of biofertilizers, therefore, the respected authors should test whether the studied bacteria could cause hypersensitivity reactions in plant models or not. Please use one of the below plants to conduct this assay. Without this validation assay, the current results cannot be published.

1- Tomato

2- Maize

3- Tobacco

4- Geraniums

5- Sugarcane

While the respected authors conduct this assay, I highly recommend you to prepare high-resolution figures of HR assays to provide detailed information for the academic readers of this journal.

**ANS:**

Thank you for your constructive suggestions.

Per your suggestions, we have performed the HR assay using tomatoes (*Solanum lycopersicum* L.) as a plant model. The method used for the HR test was injection underneath the leaf while supporting the leaf with a finger as explained by Huang et al. (1988) (lines 216-224). The HR assay result for NMS14P was negative, while *Pseudomonas aeruginosa* (positive control) caused necrotic responses on the injected leaves. The result has been incorporated into the manuscript in lines 342-344 (Results) and 647-650 (Discussion). The high-resolution figures of HR assays were provided as S3 Fig.

- **Response to the Reviewer #2**

**Reviewer #2**: The authors have sufficiently improved the manuscript. The manuscript should be accepted for publication.

**ANS:**

Thank you for your comments and suggestions to improve this manuscript.

- **Response to the Reviewer #3**

**Reviewer #3**: The authors correctly addressed all my previous comments. The manuscript is significantly improved. I have no further suggestions.

**ANS:**

Thank you for your comments and suggestions to improve this manuscript.
